# Corrigenda for four articles

**DOI:** 10.1107/S2053229615005598

**Published:** 2015-04-30

**Authors:** Qiang Li

**Affiliations:** a Ordered Matter Science Research Center, Southeast University, Nanjing 211189, People’s Republic of China

## Abstract

The acknowledgement text is corrected in four articles: Li, Wang & Ye [*Acta Cryst.* (2014), C**70**, 992–997], Li & Wang [*Acta Cryst.* (2014), C**70**, 1054–1056], Li [*Acta Cryst.* (2015), C**71**, 65–68] and Li, Wang & Zhou [*Acta Cryst.* (2015), C**71**, 93–96].

## Abstract 2

Corrigenda for four articles.

In the papers by Li, Wang & Ye (2014 ▶), Li & Wang (2014 ▶), Li (2015 ▶) and Li, Wang & Zhou (2015 ▶), the acknowledgement section is incorrect. The correct complete acknowledgement for each of the four articles is as follows:

The authors thank the College of Chemistry and Chemical Engineering, Southeast University, China, for support.
